# Analysis of the efficacy of retrodiscal approach percutaneous epidural adhesiolysis with WHIP catheter® in patients with lumbar radiculopathy: A retrospective study

**DOI:** 10.1097/MD.0000000000038452

**Published:** 2024-06-07

**Authors:** Sunmin Kim, Francis Nahm, Eun joo Cho, Pyung Bok Lee

**Affiliations:** a Department of Anesthesiology and Pain Medicine, Korea University Anam Hospital, Seoul, Korea; b Department of Anesthesiology and Pain Medicine, Seoul National University Bundang Hospital, Seongnam, Korea; c Department of Anesthesiology and Pain Medicine, Seoul National University College of Medicine, Seoul, Korea.

**Keywords:** lumbar radiculopathy, pain management, percutaneous epidural adhesiolysis, retrodiscal approach, retrospective study, WHIP catheter®

## Abstract

Percutaneous epidural adhesiolysis (PEA) is an effective treatment for patients with lumbar radiculopathy unresponsive to single steroid injections. Various approaches and instruments have been developed to access these lesions. This study aimed to evaluate the utility of a retrodiscal approach for epidural adhesiolysis using a WHIP catheter®. This retrospective study was conducted at Bundang Seoul National University Hospital, reviewing cases from January to December 2022. Forty-seven patients diagnosed with lumbar radiculopathy, aged 20 to 80 years, who underwent PEA with the WHIP catheter® were included. Outcomes assessed Numeric Rating Scale (NRS) for pain, Patients’ Global Impression of Change (PGIC) scores, and the incidence of procedure-related complications. Follow-up evaluations occurred at 1, 3, and 6 months post-procedure. Among 47 patients, 41 completed the study, showing significant pain reduction at all follow-up points: 1 month (N = 41, 1.32 ± 1.68, *P* < .001), 3 months (N = 31, 1.90 ± 2.14, *P* < .001), and 6 months (N = 30, 2.50 ± 2.30, *P* < .001). PGIC scores indicated that 40% of the patients reported substantial improvement at one-month post-procedure. The complications were minimal, with only one case of intradiscal injection and 2 cases of vascular uptake. The retrodiscal approach PEA using the WHIP catheter® demonstrated significant efficacy in pain reduction with minimal safety concerns for patients with lumbar radiculopathy. These findings suggest that this procedure is a viable option for patients who are unresponsive to conservative treatment. However, the retrospective nature of this study and its small sample size necessitate further prospective controlled studies to confirm our results and establish long-term outcomes.

## 1. Introduction

Percutaneous epidural adhesiolysis (PEA), also known as epidural lysis of adhesions or epidural neuroplasty, is a minimally invasive technique for treating cervical or lumbar radicular pain or failed back surgery syndrome that does not respond to conservative treatments.^[1]^ This procedure removes fibrous tissue (epidural adhesions) that has formed within the epidural space of the spine, thereby reducing nerve compression and playing an important role in reducing pain.^[2]^ The rationale for epidural adhesiolysis includes 2 postulated pain relief mechanisms. The first is mechanical adhesiolysis, which is achieved through the volume effect of injecting relatively large volumes of saline. This helps break up adhesions in the epidural space and flush out accumulated pain-inducing substances. The second mechanism is chemical adhesiolysis, which involves injecting therapeutic drugs such as local anesthetics and steroids. These drugs stabilize neural structures, provide analgesia, and treat inflammation of the nerves and perineural tissues.^[3]^

PEA for lumbar lesions can be performed via various routes. Typical approaches include the caudal, transforaminal, and S1 approaches,^[4]^ and a contralateral interlaminar retrograde approach has also been recently introduced.^[5]^

The transforaminal approach PEA requires a catheter specifically designed for the transforaminal approach because the space for the catheter to pass through is narrower than that in the caudal or interlaminar approach. The WHIP catheter^®^ (Mcarekorea, Seongnam, Korea) is designed to provide safe access to complex anatomical areas, especially with the recent introduction of side-hole needles to minimize potential problems, such as disc injury and intradiscal injection that can occur during the transforaminal approach.

However, well-designed studies on the overall effectiveness and safety of retrodiscal approach epidural adhesiolysis using a WHIP catheter^®^ are lacking. Therefore, this study aimed to retrospectively evaluate the clinical effectiveness and safety of epidural adhesiolysis using WHIP catheter^®^ and retrodiscal approaches.

## 2. Materials and methods

### 2.1. Study design and participants

This retrospective, single-center study was approved by the Institutional Review Board (IRB) of Bundang Seoul National University Hospital (B-2305-827-101). We identified patients who visited our hospital between January and December 2022 and reviewed their medical records. As this was a retrospective study, the requirement for informed consent was waived.

The inclusion criteria were: Patients diagnosed with lumbar radiculopathy who underwent epidural adhesiolysis using the WHIP Catheter® at a lumbar lesion. Age 20 to 80 years. Patients who had preoperative magnetic resonance imaging (MRI) of the lumbar area. The exclusion criteria were as follows: Patients who did not return for outpatient follow-up after the procedure, and Patients whose MRI findings were not correlated with their symptoms. Patients were followed up in the outpatient department of the pain clinic at 1-, 3-, and 6-months post-procedure. Numeric Rating Scale (NRS) pain scores were routinely collected during follow-up and Patients’ Global Impression of Change (PGIC) scores were obtained at 1-month follow up routinely.

### 2.2. Features of the device used

**Figure 1.**
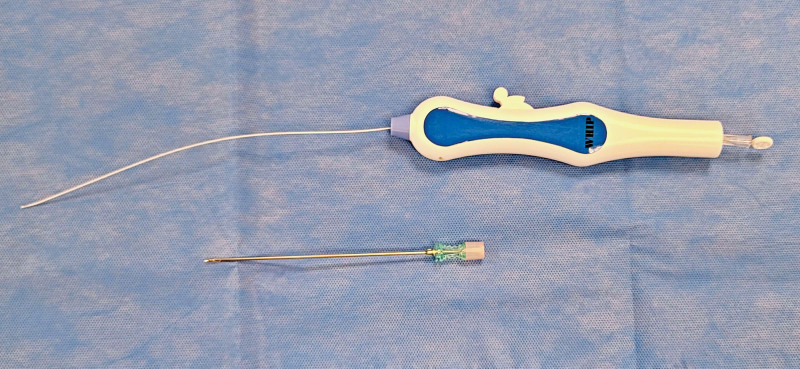
WHIP Catheter® (top) and side hole needle (bottom) for retrodiscal approach percutaneous epidural adhesiolysis.

This study used the Whip Catheter® (Mcarekorea) for retrodiscal approach epidural adhesiolysis. This set included a navigation catheter, steel guidewire, and 17G side-hole needle to perform adhesiolysis. The Whip Catheter® is a hollow catheter with a handle developed to perform epidural adhesiolysis, which allows the tip of the catheter to be bended on one side by manipulating the handle. This motion allows directional manipulation when inserting the catheter and helps perform mechanical adhesiolysis (Fig. 1). The device has a hole at the end that allows the administration of drugs or contrast media to the tip.

**Figure 2.**
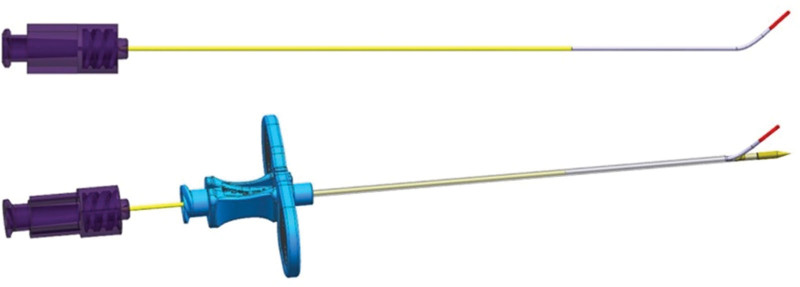
Schematic diagram of a catheter passing through a side-hole needle. The needle has a blunt tip and a side hole, with an internal slope angle allowing the catheter to smoothly advance towards the hole.

The side-hole needle is newly designed for performing retrodiscal PEA, with a length of 15 cm and 17G in outer diameter. The needle has a blunt tip and side hole, which have many advantages. Blunting the tip can reduce injury to the intervertebral disc due to disc puncture, which is a major problem with the retrodiscal approach. In addition, the side hole ensures that the catheter exiting through the needle is directed directly at the disc-nerve interface, which is the main target lesion of the procedure. Furthermore, when treating broad areas with different orientations simultaneously, the direction of the catheter could be adjusted by changing the position of the side-hole by rotating the needle (Fig. 2).

### 2.3. Interventional technique

The procedure for retrodiscal approach PEA at our center was as follows: First, the patient’s pre-procedure MRI was reviewed to determine the treatment area. The patient assumed a prone position with a pillow placed under the lower abdomen to reduce lumbar lordosis. The C-arm fluoroscope was obliquely tilted 40 to 45 degrees towards the target lesion. Typically, the C-arm fluoroscope oblique is angled obliquely to align the superior articular process below the target level with the center of the disc at the target level on the fluoroscopic image. Local anesthesia was administered at the skin entry site with 1% lidocaine, and a side-hole needle was advanced. The target of the needle was Kambin’s triangle, superior to the superior border of the vertebral body below the target level, just lateral to the superior articular process. The needle was advanced to a position where the needle tip was close to the disc on the lateral view of the fluoroscopic image or where the needle tip was felt to be in contact with the disc. Contrast medium was injected to confirm its spread into the epidural space. After confirming that the needle was in the epidural space, a whip catheter was inserted through the side-hole needle. Advance the Whip catheter to the target location while checking for fluoroscopy (commonly a lesion with a contrast filling defect). If the catheter reached the desired position, hydraluric force was used to detach the surrounding tissue using 10 cc of 0.9% normal saline containing 3000 IU of hyaluronidase. If additional target areas were present, this process was repeated. After all procedures were completed, the whip catheter was removed, and an epidural catheter was placed through the sidehole needle. Patients received 5% hypertonic saline (3 mL/h) through the epidural catheter for 2 hours, the catheter was removed, and the patient was discharged. All procedures were performed by a single pain specialist (P.B.L.).

### 2.4. Data extraction and outcomes

For this study, we collected data, including patients’ sex, age, height, weight, body mass index, spine level, direction of the procedure, and diagnostic results from MRI scans taken prior to the procedure. From the MRI diagnostic results, we specifically extracted data related to the level at which the retrodiscal approach for percutaneous epidural adhesiolysis was performed. All patient-identifying information was anonymized to ensure privacy.

The primary outcome was pain intensity using the NRS scores at 4 time points: baseline (pre-procedure) and 1, 3, and 6 months after the procedure. For the secondary outcomes, we recorded the PGIC scores, which indicated the patients’ subjective evaluation of pain, at 1-month follow-up. This was assessed using a 4-point Likert scale (1 = worsened, 2 = no change, 3 = improved, and 4 = completely improved). Additionally, we analyzed fluoroscopic images captured during the procedure to assess the presence of intradiscal injection and vascular uptake, assessing the incidence of complications during PEA. Additional procedures performed during outpatient follow-up visits were also recorded. The need for any additional procedures was determined by one pain specialist (P.B.L.).

### 2.5. Statistical analysis

Continuous variables were presented as means with standard deviations. We performed paired *t* tests to compare differences in NRS scores at each time point with baseline. Analyses were conducted using SPSS Statistics version 21 (SPSS Inc., Chicago).

## 3. Results

This study included 47 patients who were diagnosed with lumbar radiculopathy and had undergone retrodiscal approach epidural adhesiolysis using the Whip Catheter® at Seoul National University Bundang Hospital between January and December 2022. Of these, 6 patients did not return for an outpatient visit at the first 1-month follow-up, and the remaining 41 patient records were analyzed. Following this, 31 patients were analyzed at the next 3-months and 30 at 6-months. The study comprised 26 males (63.5%) and 15 females (36.5%). Participants had an age as a minimum of 40 and a maximum of 76 with a mean age was 67.00 ± 10.71. The most common diagnosis in the study population was herniated intervertebral disc (HIVD) in 33 patients (80.4%), and foraminal stenosis was the most common subtype in 18 patients. The second most common diagnosis was failed back surgery syndrome in 8 patients (19.5%), spondylisthesis in 2 patients (4.8%), and compression fracture in 1 patient (2.4%). Three patients had multiple diagnoses: 1 patient with HIVD mixed type and spondylolisthesis, 1 patient with HIVD mixed type and facet arthrosis, and 1 patient with HIVD central stenosis and facet arthrosis (Table 1).

**Table 1:** Characteristics of all enrolled participants.

Characteristics	Mean ± SD, N (%)
Age	67.00 ± 10.71
Sex	M: 26, F: 15
Male	26 (63.4)
Female	15 (36.6)
Height	163.20 ± 10.02
Weight	66.06 ± 12.30
Body mass index	24.67 ± 12.53
Diagnosis	
HIVD	33 (73.3)
Central stenosis	10 (22.2)
Foraminal stenosis	18 (40.0)
Mixed	5 (11.1)
FBSS	8 (17.8)
Spondylolisthesis	2 (4.4)
Facet joint arthosis	1 (2.2)
Compression fracture	1 (2.2)
Procedure orientation	
Right	22 (53.7)
Left.	19 (46.3)
Procedure level	
L4	27 (65.9)
L5	14 (34.1)

Values are presented as mean ± standard deviation or numbers and percentage.

CRPS = complex regional pain syndrome, FBSS = failed back surgery syndrome, HIVD = herniated intervertebral disc, PHN = post herpetic neuralgia, SD = standard deviation.

**Figure 3.**
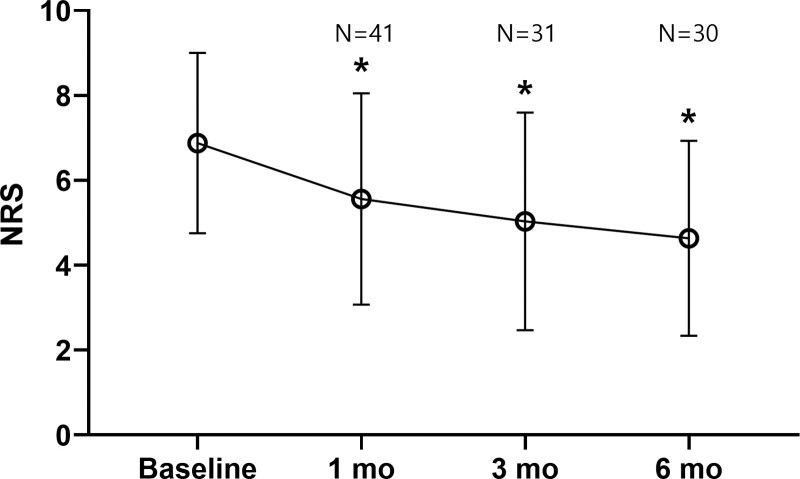
Changes in the Numeric Rating Scale (NRS) of pain at baseline and at 1, 3, and 6 months. At baseline and the 1-month follow-up, 41 patients were included, with the number decreasing to 31 at the 3-month follow-up and to 30 at the 6-month follow-up due to loss of follow-up. The average NRS showed a continuous decrease. *Significant at *P* < .05, compared to the baseline NRS.

The degree of pain reduction on the NRS was compared between baseline and 1-month post-procedure outpatient follow-up. A total of 41 patients were included, with a mean reduction in pain of 1.32 ± 1.68 (*P* < .001). When comparing baseline to 3 months, 31 patients were included, excluding 10 patients with missing follow-up data, with a mean pain reduction of 1.90 ± 2.14 (*P* < .001). When comparing the NRS from baseline to 6 months, 30 patients were included, showed a mean pain reduction of 2.50 ± 2.30 (*P* < .001). At all time points, the NRS was reduced statistically significantly compared to baseline (Fig. 3).

**Figure 4.**
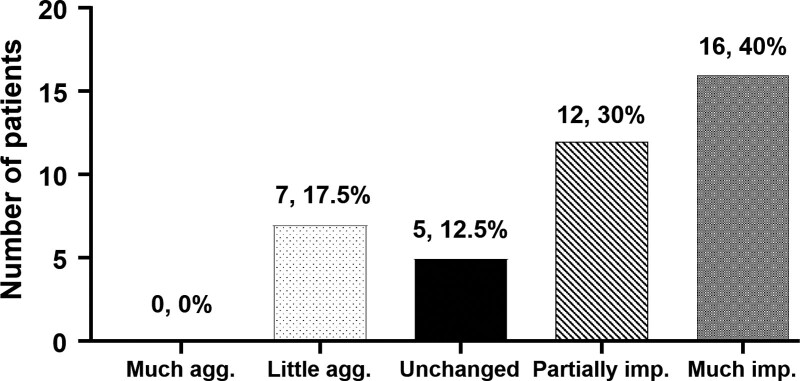
The PGIC scores, which were obtained at the first outpatient follow-up at 1 month after the procedure. PGIC = The Patients’ Global Impression of Change scores.

The PGIC scores, which were obtained at the first outpatient follow-up 1 month after the procedure, were as follows: 16 patients (40%) reported much improved, 12 patients (30%) reported partially improved, 5 patients (12.5%) reported unchanged, and 7 patients (17.5%) reported more aggravated. One patient was excluded because the response was not included in the medical records (Fig. 4).

In the fluoroscopic image analysis, intradiscal injection occurred in one (2.5%) patient who was diagnosed with a compression fracture and underwent the procedure, and vascular uptake was observed in the fluoroscopic images of 2 (5%) patients who were diagnosed with failed back surgery syndrome and HIVD mixed stenosis.

A total of 24 (58.5%) patients underwent additional procedures at outpatient follow-up after retrodiscal PEA. Additional procedures are commonly carried out during the pain center’s treatment process when pain interventions are insufficient or ineffective. The procedures are grouped into similar categories (Table 2). Five patients underwent multiple procedures: 3 with trigger point injection (TPI) and transforaminal epidural steroid injections, 1 with TPI and facet joint injection, and 1 with TPI and transcutaneous electrical nerve stimulation.

**Table 2:** Additional procedures performed during 6-month outpatient follow-up.

Additional procedures	Patients (N, %)
None	17 (40.0)
TPI, IMS, Needle Tens	13 (28.3)
Epidural steroid injection	8 (17.4)
Facet joint injection	3 (6.5)
MBB	1 (2.1)
TENS, Scrambler therapy	4 (8.7)
Total	46

IMS = intramuscular stimulation, MBB = medial branch block, TENS = Transcutaneous Electrical Nerve Stimulation, TPI = trigger point injection.

## 4. Discussion

The theoretical basis of PEA lies in the presence and dissolution of pain-induced epidural adhesions. This interventional technique was developed in the late 1980s by Racz et al.^[6,7]^ Since it was introduced, percutaneous adhesiolysis has been successfully used to treat low back and lower extremity pain that does not resolve with conventional mordalities such as epidural steroid injections.^[8–10]^

Various approaches exist for performing epidural adhesiolysis using a catheter. When this technique was first introduced in the literature, an approach using the caudal canal was described.^[7,11]^ However, when the targeted levels are higher than L5/S1, access from the caudal canal is very time-consuming because of the difficulty in handling the catheter. Furthermore, passage of the epidural catheter may be challenging if degenerative changes occur in the spinal canal.^[12]^ For these reasons, approaches from different sites have been studied. Currently, lumbar PEAs are generally performed using the caudal, transforaminal, and S1 approaches. In a study by Salem et al, no significant difference was found among these 3 approaches in reducing pain and improving function in patients with post-lumbar surgery syndrome.^[4,13]^ Recently, a contralateral interlaminar retrograde approach has been introduced.^[5]^

When performing PEA via a transforaminal approach, 2 options are available: an approach through the safe triangle and an approach through the retrodiscal space. The safe triangle is a foramen formed by the diagonal path of the nerve, the base of the pedicle, and the lateral border of the vertebral body; it is called the “safe triangle” because no nerves pass inside this space. Above this pathway, however, is the segmental medullar artery, represented by the Adamkiwiecs artery.^[14]^ Although there have been no reports of complications during percutaneous epidural adhesiolysis through this pathway, there have been several case reports of spinal cord ischemia due to injury to the segmental medullar artery during epidural steroid injection through this pathway.^[15–17]^ Percutaneous epidural adhesiolysis requires the use of a thicker needle and more varied instruments than transforaminal epidural steroid injections. Thus, the retrodiscal approach may be the preferred option among the 2 pathways to minimize the risk of these severe complications. Gil et al^[18]^ also compared pain reduction on the visual analog scale, improvement of function in the Oswestry disability score, and the incidence of complications between these 2 approaches and showed no difference between the 2 groups.

Several types of catheters are used to treat percutaneous epidural adhesiolysis. In addition to the Racz catheter, which was the first to be developed, other options include nerve stimulation catheters (EpiStim® catheter; Sewoon Medical, Cheonan, Korea), steerable navigation catheters (NaviCath®; Myelotec, Roswell), zigzag-motion inflatable neuroplasty catheters (ZiNeu®; JUVENUI, Seongnam, Korea).^[19]^ However, only a few comparative studies have been conducted on the effectiveness of these catheters. In a study by Karm et al, which compared the effectiveness of an inflatable balloon catheter and a non-balloon catheter when performing PEN in patients with central lumbar spinal stenosis, the balloon appeared to be superior to the non-balloon catheter.^[20]^ This seems obvious, given that a catheter with a balloon physically detaches adhesions more effectively. However, this study investigated only the caudal approach and not the transforaminal approach. In addition, the study by Karm et al used the ZiNeu® catheter as an inflatable balloon catheter, which can only be used for the caudal approach. None of the inflatable catheters used in the zigzagging motion were developed for the transforaminal approach, which made it difficult to place the catheter in the target area.

The transforaminal approach using a Whip catheter®, which we used in our study, has several advantages. While it does not provide physically strong epidural adhesiolysis, as with an inflatable balloon catheter, it allows directional movements to access the target lesion, and can use a thinner gauge needle, potentially preventing complications. Additionally, the Whip catheter® uses a side-hole needle that has a blunt tip. The reason for adopting a blunt tip for this needle was to reduce inadvertent intradiscal and intravascular injections, which are common complications during retrodiscal approach procedures. While a blunt tip can make it more challenging to penetrate relatively tense skin, this feature helps prevent disc puncture by conveying the sensation of the needle touching the disc surface to the practitioner. According to a study by Levi et al, intradiscal injection was retrospectively observed in 8 of 257 cases (3.1%) during Retrodiscal Approach-Transforaminal Epidural Steroid Injection using a 22G or 25G Quincke needle.^[21]^ Additionally, a retrospective study by Lee et al reported intradiscal injection in 6 of 106 cases (5.7%) of Retrodiscal Approach-Transforaminal Epidural Steroid Injection using a 20G Tuohy needle.^[22]^ Inadvertent intradiscal injections not only inflict mechanical damage to the disc but also precipitate discitis, a serious complication. This condition demonstrates marked resistance to standard treatments and carries the risk of permanent neurological sequelae.^[23]^ The incidence of discitis when the block needle enters the disc is unknown, but the incidence of discitis after cervical discography has been reported to be between 0.15% and 0.44% and, after lumbar discography, between 0.00% and 4.92%.^[24,25]^ In our study, the use of these needles resulted in a single case (2.5%) of inadvertent intradiscal injection.

Our study shows the potential benefits of the retrodiscal approach percutaneous epidural adhesiolysis with the Whip catheter®. The unique design of this catheter, particularly its side-hole needle, provides more directed treatment at the disc-nerve interface, which is crucial in managing lumbar radiculopathy. This study demonstrated significant pain reduction in patients over a 6-month follow-up period, emphasizing the clinical efficacy of this approach. However, acknowledging the limitations of the Whip catheter® is crucial. First, when using a transforaminal approach, the design of this catheter may not be as effective in treating multiple lesions simultaneously because it is difficult to access lesions at levels beyond the initial point of entry or on the opposite side. The focused approach of the Whip catheter®, while beneficial in targeting specific areas, can be a drawback when dealing with widespread or multiple spinal lesions. Second, while this study showed promising results, it is important to consider that the effectiveness of the Whip catheter® may not match that of the inflatable catheters. Although no direct research has compared these 2 techniques, the mechanical advantage provided by inflatable catheters in terms of physically breaking down adhesions could potentially offer more significant pain relief. However, this hypothesis must be tested in future studies.

Our study had some limitations. The first is the retrospective design without a control group, which limits the ability to make definitive conclusions about the efficacy of the Whip catheter® compared to other treatment modalities. This design is inherently biased and restricts comparative analysis. Additionally, the study’s follow-up duration was limited to 6 months. Although we observed significant improvements during this period, the frequent need for additional procedures suggests that the long-term benefits of this approach may be limited. A longer follow-up period is necessary to evaluate the sustained efficacy of the Whip catheter® and its long-term effects on patient outcomes.

## 5. Conclusion

In conclusion, our study provides preliminary evidence suggesting the potential benefits of the retrodiscal approach to percutaneous epidural adhesiolysis using the Whip catheter® for treating lumbar radiculopathy. However, given the highlighted limitations, including the potential for lower effectiveness in treating multiple lesions and the absence of long-term follow-up data, further research is essential. Prospective studies with control groups and extended observation periods are critical to validating and expanding these findings.

## Author contributions

**Conceptualization:** Eun joo Choi, Pyung Bok Lee.

**Data curation:** Sunmin Kim.

**Formal analysis:** Sunmin Kim.

**Investigation:** Eun joo Choi.

**Methodology:** Francis Nahm.

**Software:** Francis Nahm.

**Supervision:** Eun joo Choi, Pyung Bok Lee.

**Writing – original draft:** Sunmin Kim.

**Writing – review & editing:** Pyung Bok Lee.

## References

[1] Lee F Jamison DE Hurley RW Cohen SP. Epidural lysis of adhesions. Korean J Pain. 2014;27:3–15. 24478895 10.3344/kjp.2014.27.1.3 PMC3903797

[2] Jamison DE Hsu E Cohen SP. Epidural adhesiolysis: an evidence-based review. J Neurosurg Sci. 2014;58:65–76. 24819483

[3] Choi EJ Yoo YJ Lee PB Kim YC Lee SC Moon JY. A retrospective study to evaluate the effect of concentration of hypertonic saline on efficacy and safety of epidural adhesiolysis. Anesth Analg. 2017;124:2021–9. 28448392 10.1213/ANE.0000000000001925

[4] Salem HH. Comparison of 3 approaches to percutaneous epidural adhesiolysis and neuroplasty in post lumbar surgery syndrome. Pain Physician. 2018;1:E501–8. 30282398

[5] Kim CS Moon YJ Kim JW. Transforaminal epidural balloon adhesiolysis via a contralateral interlaminar retrograde foraminal approach: a retrospective analysis and technical considerations. J Clin Med. 2020;9:981. 32244742 10.3390/jcm9040981 PMC7230206

[6] Manchikanti L Bakhit CE. Percutaneous lysis of epidural adhesions. Pain Physician. 2000;3:46–64. 16906207

[7] Racz GB Holubec JT. Lysis of adhesions in the epidural space. In: Racz GB, ed. Techniques of Neurolysis. Springer; 1989:57–72.

[8] Helm S. Percutaneous and endoscopic adhesiolysis in managing low back and lower extremity pain: a systematic review and meta-analysis. Pain Physician. 2016;19:E245–81. 26815254

[9] Gerdesmeyer L Wagenpfeil S Birkenmaier C. Percutaneous epidural lysis of adhesions in chronic lumbar radicular pain: a randomized, double-blind, placebo-controlled trial. Pain Physician. 2013;16:185–96. 23703406

[10] Aoi I Chen HC Lui TN Lin TJ. Outcomes of epidural neuroplasty utilizing adhesiolysis by means of hydraulic and mechanical force. J Biomed. 2018;3:26–31.

[11] Racz GB Heavner JE Prithvi Raj P. Epidural neuroplasty. Semin Anesth Perioper Med Pain. 1997;16:302–12.

[12] Kim JS Lee JH Ahn Y, eds. Endoscopic Procedures on the Spine. Springer; 2020.

[13] Shin JW. Effects of transforaminal balloon treatment in patients with lumbar foraminal stenosis: a randomized, controlled, double-blind trial. Pain Physician. 2013;3;16:213–24. 23703408

[14] Taterra D Skinningsrud B Pękala PA. Artery of adamkiewicz: a meta-analysis of anatomical characteristics. Neuroradiology. 2019;61:869–80. 31030251 10.1007/s00234-019-02207-y PMC6620248

[15] Lyders EM Morris PP. A case of spinal cord infarction following lumbar transforaminal epidural steroid injection: MR imaging and angiographic findings. AJNR Am J Neuroradiol. 2009;30:1691–3. 19369604 10.3174/ajnr.A1567 PMC7051528

[16] Kennedy DJ Dreyfuss P Aprill CN Bogduk N. Paraplegia following image-guided transforaminal lumbar spine epidural steroid injection: two case reports. Pain Med. 2009;10:1389–94. 19863744 10.1111/j.1526-4637.2009.00728.x

[17] Wang G Liang J Jia Z Wan L Yang M. Spinal cord infarction caused by sacral canal epidural steroid injection: a case report. Medicine (Baltimore). 2018;97:e0111. 29538204 10.1097/MD.0000000000010111 PMC5882383

[18] Gil HY Jeong S Cho H Choi E Nahm FS Lee PB. Kambin’s triangle approach versus traditional safe triangle approach for percutaneous transforaminal epidural adhesiolysis using an inflatable balloon catheter: a pilot study. J Clin Med. 2019;8:E1996. 10.3390/jcm8111996 PMC6912526 31731783

[19] Kim DH Shin JW Choi SS. Percutaneous epidural balloon neuroplasty: a narrative review of current evidence. Anesth Pain Med (Seoul). 2022;17:361–70. 36317428 10.17085/apm.22237 PMC9663944

[20] Karm MH Choi SS Kim DH. Percutaneous epidural adhesiolysis using inflatable balloon catheter and balloon-less catheter in central lumbar spinal stenosis with neurogenic claudication: a randomized controlled trial. Pain Physician. 2018;21:593–606. 30508987

[21] Levi D Horn S Corcoran S. The incidence of intradiscal, intrathecal, and intravascular flow during the performance of retrodiscal (infraneural) approach for lumbar transforaminal epidural steroid injections. Pain Med. 2016;17:1416–22. 26814293 10.1093/pm/pnv067

[22] Lee J Jo D Song S Park D Kim D Oh J. Effect of needle tip position on contrast media dispersion pattern in transforaminal epidural injection using Kambin’s triangle approach. J Pain Res. 2020;13:2869–78. 33204148 10.2147/JPR.S270450 PMC7667514

[23] Cohen SP Larkin TM Barna SA Palmer WE Hecht AC Stojanovic MP. Lumbar discography: a comprehensive review of outcome studies, diagnostic accuracy, and principles. Reg Anesth Pain Med. 2005;30:163–83. 15765459 10.1016/j.rapm.2004.10.006

[24] Guyer RD Ohnmeiss DD Vaccaro A. Lumbar discography. Spine J. 2003;3(3 Suppl):11–27. 14589214 10.1016/s1529-9430(02)00563-6

[25] Kapoor SG Huff J Cohen SP. Systematic review of the incidence of discitis after cervical discography. Spine J. 2010;10:739–45. 20171935 10.1016/j.spinee.2009.12.022

